# MicroRNA expression levels as diagnostic biomarkers for intraductal papillary mucinous neoplasm

**DOI:** 10.18632/oncotarget.17679

**Published:** 2017-05-08

**Authors:** Lei Wang, Jianming Zheng, Chang Sun, Li Wang, Gang Jin, Lei Xin, Zhendong Jin, Dong Wang, Zhaoshen Li

**Affiliations:** ^1^ Department of Gastroenterology, Changhai Hospital, Second Military Medical University, Shanghai 200433, China; ^2^ Department of Pathology, Changhai Hospital, Second Military Medical University, Shanghai 200433, China; ^3^ Department of Radiology, Changhai Hospital, Second Military Medical University, Shanghai 200433, China; ^4^ Department of General Surgery, Changhai Hospital, Second Military Medical University, Shanghai 200433, China

**Keywords:** intraductal papillary mucinous neoplasms (IPMNs), miRNA, cancer diagnosis

## Abstract

Intraductal papillary mucinous neoplasms (IPMNs) are deadly exocrine mucinous tumors. Currently the molecular features and diagnostic markers of IPMNs are not well understood. In this study, we performed microRNA (miRNA) profiling assays to study the potential roles of miRNAs in IPMNs using 78 cases of IPMN patients and controls. When comparing the miRNA expression between IPMN patient samples and controls, we found that miR-210, miR-223, miR-221, miR-155 and miR-187 were differentially expressed in normal pancreas and IPMNs. We further studied the miRNA expression profiles in different pancreatic diseases and identified miRNA features that are associated with Chronic pancreatitis (CP), IPMN, and Pancreatic ductal adenocarcinoma (PDAC). Therefore, these miRNAs might serve as new risk biomarkers of IPMN and could be useful for future targeted therapies.

## INTRODUCTION

Intraductal papillary mucinous neoplasms (IPMNs) are a class of visceral exocrine mucinous tumors, ranging from benign adenoma to infiltrative carcinoma [1]. IPMNs can be divided into benign (adenoma), borderline, malignant noninvasive (carcinoma *in situ*) and invasive carcinoma (IC-IPMC, infiltrating duct papillary mucinous carcinoma), depending on whether the infiltrating basement membrane and different cell morphology and histological structure [2]. The IC-IPMC is a very deadly cancer, the 3, 5, 10-year survival rate of IC-IPMC patient is 51%, 38% and 0%, respectively [3, 4]. Therefore, development of novel, effective diagnostic markers and therapeutic targets for IPMNs is in urgent need for the better treatment of IPMN patients.

MicroRNAs (miRNAs) are small, non-coding RNA molecules participating in post-transcriptional gene regulation and have been identified to be involved in cancer development [5–9]. MiRNAs are frequently mis-regulated in cancers: some miRNAs are lowly expressed in tumor tissues and function as tumor suppressors. On the other hand, some miRNAs are highly expressed in tumor tissues and play the roles as oncogenes to promote oncogenesis [7–10]. Because of their stable nature and the circulating features, miRNAs have been widely used for cancer diagnostics and treatments [11–14]. Importantly, alterations in miRNAs expression have been related to cancer pathogenesis in IPMNs [15, 16]. Here we studied the differential miRNA expression between benign and malignant IPMN patients and have identified several miRNAs that could serve as novel diagnostic markers for malignant IPMNs.

## RESULTS

### Clinicopathologic features of the IPMN patient samples

We first determined the clinicopathologic features of the IPMN patient samples using the well known biochemical indicators and tumor markers (Table 1). Comparing the levels of the above indicators, we found that only total bilirubin (T-Bil) and CA19-9 levels are significantly different between the benign and malignant IPMN patient samples, indicating the possible roles of these two factors in the pathogenesis of IPMNs. Indeed, Serum CA19-9 level can be used as a non-invasive preoperative tool to distinguish invasive and benign IPMNs [17], which is consistent with our result. For all other indicators or tumor markers examined in our study, where were no significant difference in their levels between benign and malignant IPMN patient samples, which suggests that the current well known indicators or tumor markers are not effective for the diagnostics of IPMNs.

**Table 1:** Clinicopathologic features of the IPMN patient samples

Index	Benign			Malignant			p-value
	Case no	Mean	SD	Case no		SD	
**Tbil**	38	13.412	15.129	40	35.053	62.485	0.039
**DBil**	38	6.202	11.557	40	19.610	41.526	0.056
**ALB**	38	39.740	3.922	40	39.547	4.173	0.834
**ALT**	38	40.607	54.439	39	59.324	81.164	0.240
**AST**	38	31.685	19.672	39	49.732	54.694	0.059
**AKP**	38	98.549	145.348	39	174.979	204.390	0.062
**GGT**	37	93.240	312.035	39	207.966	377.085	0.152
**GLU**	37	5.390	1.852	39	5.176	1.122	0.545
**TG**	10	2.518	1.789	12	31.220	99.474	0.374
**CH**	10	2.536	0.981	12	3.376	1.370	0.121
**AMY**	20	133.475	193.409	12	61.800	69.425	0.128
**CEA**	33	2.222	1.219	12	30.470	99.770	0.094
**CA19-9**	34	22.847	38.078	12	141.576	244.062	0.008

### MiRNA expression profiles in IPMN patient samples

**Figure 1:**
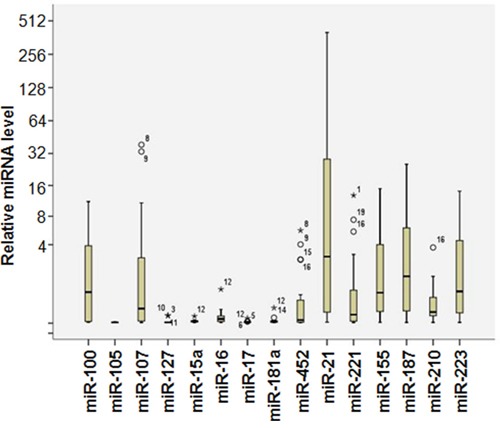
MicroRNA expression profiles in IPMN patient samples miRNA expression levels were determined by SYBR Green I base quantitative PCR. Briefly, RNA samples were *in vitro* transcribed in to cDNA using miRNA specific stem-loop RT primers, the the RT products were used for the real time PCR analysis. The expression of individual miRNAs were normalized to Let-7a using 2^-ΔCt^ method. N=3.

We next investigated whether miRNAs could serve as diagnostic markers for the IPMNs. First we performed a miRNA profiling study to detect the expression of 15 pancreas related miRNAs that could potentially be mis-regulated in IPMNs [18–20]. We found that different miRNAs have different expression levels in the patient samples. As shown in Figure 1, some miRNAs (miR-17, miR-107) have very low expression, while on the other hand, miRNAs such as miR-21 have very strong expression levels. These data suggested that we can successfully detect the miRNA expression and different miRNAs are differentially expressed in the IPMN patient samples.

### Mis-regulated miRNA expression in IPMN patients

**Figure 2:**
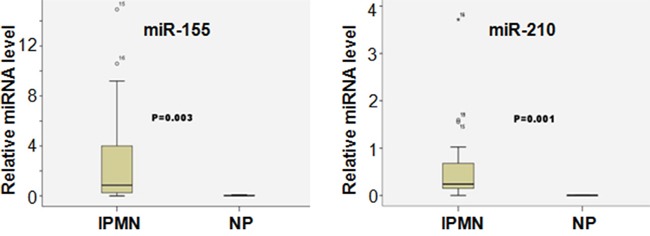
Differential miRNA expression between IPMN and NP samples The relative expression levels of miR-155 and miR-210 were determined by real time PCR analysis using IPMS and NP samples.

To further study the potential role of miRNAs in the pathogenesis of IPMNs, we determined the expression of individual miRNAs in IPMN patient samples and the normal control pancreas (NP). As shown in Table 2 and Figure 2, among the 15 miRNAs examined in our study, 9 of them (miR-210, miR-210, miR-223, miR-221, miR-221, miR-221, miR-155, miR-210, miR-223) were significantly differentially expressed between the IPMN samples and the controls. Interestingly, all of those 9 miRNAs were up-regulated in the IPMN patients compared to controls, suggesting that those miRNAs could potentially promote the pathogenesis of IPMNs.

**Table 2:** Mis-regulated miRNA expression in IPMN patients

miRNAs	IPMN			NP			p-value
	Mean	Quartiles		Mean	Quartiles		
**miR-100**	0.490	0.016	3.491	0.006	0.004	0.010	.002
**miR-107**	0.298	0.024	2.757	0.004	0.002	0.013	.004
**miR-452**	0.045	0.002	0.582	0.001	0.000	0.002	.005
**miR-21**	2.539	0.236	25.642	0.159	0.079	0.334	.018
**miR-221**	0.162	0.017	0.871	0.002	0.000	0.007	.002
**miR-155**	0.807	0.178	3.812	0.024	0.018	0.051	.003
**miR-187**	1.315	0.192	5.399	0.002	0.001	0.006	.001
**miR-210**	0.220	0.137	0.600	0.002	0.001	0.004	.001
**miR-223**	0.739	0.202	4.178	0.002	0.001	0.005	.000

### Differential expression of miRNAs in different IPMN subtypes

We further studied the expression profile of miRNAs in IPMNs, we detected the expression of different miRNAs in different IPMN subtypes. IPMN samples were separated into four different subtypes (G, gastric type; I, intestinal type; PB, biliary pancreatic type; O: oncocytic type). The G and I subtypes of IPMNs have relative good prognosis, while the PB and O types of IPMN represent the poor prognosis types. When we compared the miRNA expression levels in different IPMN subtypes, we found that, interestingly, four miRNA (miR-100, miR-187, miR-210, miR-223) levels were up-regulated in the poor prognosis subtypes (PB and O) compared to the good prognosis subtypes (G and I) (Table 3), suggesting that the expression levels of those miRNAs were associated with the IPMN prognosis statuses.

**Table 3:** Differential expression of miRNAs in different IPMN subtypes

miRNAs	G+I			PB+O			p-value
	Mean	Quartiles		Mean	Quartiles		
**miR-100**	0.096	1.384	1.939	2.281	1.384	6.539	0.086
**miR-187**	0.431	0.115	3.330	2.654	1.816	15.591	0.086
**miR-210**	0.194	0.110	0.304	0.678	0.318	1.288	0.037
**miR-223**	0.447	0.120	2.211	3.418	0.740	6.595	0.052

### Differential miRNA expression between chronic pancreatitis (CP), IPMN, and pancreatic ductal adenocarcinoma (PDAC) samples

The mis-regulated miRNA expression in IPMN suggests the important role of miRNA in IPMN prognosis. Next we studied the expression of miRNAs between different pancreatic diseases. The process of carcinogenesis of PDAC is usually considered as a cumulative mutation process that first started with chronic pancreatitis and later tumor development. We first compared the expression of miRNAs between IPMN and CP samples (Table 4), and found that the expression of miR-21, miR-155, miR-187, miR-210 and miR-223 are significantly higher in IPMN patient samples compared to the CP samples. When comparing the IPMN with PDAC samples (Table 5), we identified four different miRNAs, miR-16, miR-17, miR-181a and miR-187, that are differentially expression between the IPMN and PDAC samples. The above differentially expressed miRNAs between CP, IPMN and PDAC samples could serve as novel diagnostic markers for different pancreatic diseases.

**Table 4:** Differential miRNA expression between chronic pancreatitis and IPMN patient samples

miRNAs	IPMN			CP			p-value
	Mean	Quartiles		Mean	Quartiles		
**miR-21**	2.539	0.236	25.642	0.161	0.109	1.105	0.049
**miR-155**	0.807	0.178	3.812	0.070	0.024	0.086	0.006
**miR-187**	1.315	0.192	5.399	0.008	0.002	0.232	0.014
**miR-210**	0.220	0.137	0.600	0.010	0.002	0.038	0.003
**miR-223**	0.739	0.202	4.178	0.051	0.006	0.324	0.039

**Table 5:** Differential miRNA expression between PDAC and IPMN patient samples

miRNAs	IPMN			PDAC			p-value
	Mean	Quartiles		Mean	Quartiles		
**miR-16**	0.063	0.011	0.140	0.157	0.081	0.272	0.028
**miR-17**	0.007	0.004	0.009	0.019	0.009	0.033	0.005
**miR-181a**	0.021	0.001	0.030	0.073	0.018	0.182	0.008
**miR-187**	1.315	0.192	5.399	0.170	0.061	0.811	0.010

## DISCUSSION

MiRNAs are 18~24 nucleotides, non-coding small RNAs that plays important roles in tumorigenesis, development, prognosis, diagnosis and treatment responses. At post-transcriptional level, it binds to the target mRNA, and negatively regulates the expression of the target genes [7]. Recent studies have shown that miRNAs can target important target genes or pathways and play essential roles as oncogenes or tumor suppressor genes in tumorigenesis and progression [21–23], therefore, miRNA could serve as important cancer diagnostic markers and therapeutic targets.

MiRNAs are widely used in the diagnosis of pancreatic cancers. Recent studies revealed that miRNA expressions in pancreatic cancer samples, para-cancerous tissues, normal tissues and chronic pancreatitis were significantly different, suggesting that these differentially expressed miRNAs may be able to detect and treat the pancreatic diseases [20, 24–26]. Our results revealed that miR-100, miR-107, miR-215, miR-21, miR-221, miR-155, miR-187, miR-210 and miR-223 are differentially expressed between normal pancreas and IPMNs, suggesting that these miRNAs may be specific markers of IPMN. Our profiling data were consistent with some published data reporting the important roles of the above miRNA in pancreatic diseases. For example, miR-155 can inhibit the expression of Tumor protein 53-induced nuclear protein 1 expression and therefore promote pancreatic tumor development [16, 25, 27]. And miR-210 has been identified as a biomarker of pancreatic ductual adenocarcinoma [28]. In addition, the expression of miR-21, miR-155, miR-187, miR-210 and miR-223 in are significantly different in IPMN and CP patients and the expression of miR-16, miR-181a and miR-187 in were differentially expressed in between IPMNs and PDACs, suggesting that these miRNAs could be used as detection markers for different pancreatic diseases.

Overall, our data suggested that miRNA are important regulators of IPMN and other pancreatic diseases. The differentially expressed miRNAs could potentially be used as novel diagnostic markers and therapeutic targets for the treatment of IPMNs.

## MATERIALS AND METHODS

### Patients and clinical samples

78 cases of IPMN patients treated in Shanghai Changhai Hospital during the period from 2000-2009 were used for this study. The case group consisted of patients with benign IPMN (n=38) and patients with malignant IPMN (n=40). Patient samples were tested using different markers including: serum total protein (TP), albumin (Alb), total bilirubin (T-Bil), alanine transferase (ALT), aspartate transferase (AST) and the tumor markers carbohydrate antigen (CA19-9). The postoperative serum levels of ALP, γ-glutamyltransferase (γ-GTP), amylase (Amy), carcinoembryonic antigen (CEA). The pathological statuses of the samples were defined with WHO classification criteria. Written informed consents were signed by all the patients before collecting samples. The study protocol was approved by the Institutional Ethic Committee of the Hospital.

### Isolation of miRNA from formalin-fixed paraffin-embedded tissues

MiRNA samples were isolated from Formalin-Fixed Paraffin-Embedded Tissues using the Roche miRNA isolation kit. Briefly, the paraffin specimens were cut into 5μm thick slices and then treated with xylene for 5 minutes at room temperature, then RNA the precipitated using pure ethonal. MiRNA samples were further isolated using the RNA binding column following the manufacturer's instructions.

### MiRNA expression analysis

MiRNA expression was determined using the previously established SYBR Green I based method [29]. The pancreatic paraffin tissue small RNAs were reverse transcribed (RT) into cDNA using different miRNA stem-loop RT primers. Then the RT products were diluted 5 times using H_2_O and used for the Real time PCR analysis. Let-7a was used as internal control. The relative expression levels of miRNAs were calculated using the 2^-ΔCt^ method. The presented data were from three independent repeats for each sample.

### Statistical analysis

SPSS 13.0 statistical software was used for statistical processing and statistical charts. Since the miRNA data are non-normally distributed, the expression data are expressed as median and interquartile range. Mann-Whitney U test (two groups) and Kruskal-Wallis H test (multiple groups) were used for statistical analysis, p<0.05 for the hypothesis test was considered as statistically significant.
